# Ecological parameters of the (S)-9-methylgermacrene-B population of the *Lutzomyia longipalpis* complex in a visceral leishmaniasis area in São Paulo state, Brazil

**DOI:** 10.1186/s13071-017-2211-8

**Published:** 2017-05-30

**Authors:** Fredy Galvis-Ovallos, Claudio Casanova, Anaiá da Paixão Sevá, Eunice Aparecida Bianchi Galati

**Affiliations:** 1 Programa de Pós-Graduação em Saúde Pública, Faculdade de Saúde Pública, Universidade São Paulo - USP, São Paulo, SP Brazil; 2 Superintendência de Controle de Endemias, Secretaria de Estado da Saúde, Mogi Guaçu, São Paulo Brazil; 3 Programa de Pós-Graduacão em Epidemiologia Experimental Aplicada às Zoonoses, Faculdade Medicina Veterinária, Universidade de São Paulo-USP, São Paulo, SP Brazil; 4 Departamento de Epidemiologia, Faculdade de Saúde Pública, Universidade São Paulo - USP, São Paulo, SP Brazil

## Abstract

**Background:**

Visceral leishmaniasis (VL) is an important public health challenge in Brazil because of the high number of human and canine cases reported annually. *Leishmania infantum* is the etiological agent of VL and *Lutzomyia longipalpis* is its main vector. However, evidence suggests that this taxon constitutes a species complex. In Sao Paulo state, there are two populations of *Lu. longipalpis*, each secreting distinct pheromones, (S)-9-methylgermacrene-B and Cembrene 1; both have been associated with different patterns of VL transmission. The aim of the present study was to investigate the temporal distribution and natural infection of the (S)-9-methylgermacrene-B population of the *Lu. longipalpis* complex in a highly VL endemic area of Sao Paulo state to obtain information that may contribute to the surveillance of this zoonosis and to the planning of preventive and control measures.

**Methods:**

The study was carried out in Panorama municipality, Sao Paulo State. Captures were made during 24 months in seven domiciles. The relation between sand fly abundance and climatic variables, temperature and humidity, was analyzed and natural infection by *Leishmania* spp. in sand fly females was investigated by nested PCR.

**Results:**

A total of 4120 sand flies, with predominance of *Lu. longipalpis* (97.2%) were captured. The highest averages of sand flies/night/trap occurred in the rainy season (November-March) and a positive, significant correlation between sand fly abundance and the temperature and humidity 20 days before the capture days was found. *Leishmania infantum* DNA was detected in three out of 250 pools of females analyzed, giving an estimated minimum infection rate of 1.2%.

**Conclusion:**

The identification of the climatic association between the high abundance of the vector in this highly endemic VL focus constitutes a fundamental point for evaluating future vector and dog control measures and this information increases the data of VL foci in Sao Paulo state that could contribute to the public health authorities in planning prevention and control measures. The identification of natural infection by *Le. infantum* in *Lu. longipalpis* specimens reinforces the importance of entomological surveillance activities in this municipality.

## Background

The phlebotomine sand flies (Diptera: Psychodidade) are of epidemiological importance due to the involvement of several species in the transmission of pathogenic agents of diseases such bartonelloses, arboviruses and mainly the leishmaniasis [1, 2]. From 1999 to 2013, there were recorded in Brazil 53,067 new cases of visceral leishmaniasis (VL), with an annual mean of 3500 new cases. From 1999 to 2013 São Paulo state, recorded 2328 new cases of the disease distributed in 80 municipalities, with 202 deaths [3]. Of the 520 existing American sand flies described [4], *Lutzomyia longipalpis* is the Neotropical species most widely studied because of its epidemiological importance as the main vector of *Leishmania infantum*, agent of VL [5–7]. This species occur from Mexico to Argentina, with the exception of Ecuador, Peru and Chile, and is widely distributed in Brazil where has shown in recent years that it is well adapted to urban areas [8]. Undoubtedly, this fact has further contributed to its rapid expansion [6–9] and constitutes a challenge to VL control programs [7, 10]. Until 2015, *Lu. longipalpis* has been reported in 177 municipalities of São Paulo state [11]. However ecological, biochemical and genetic evidences suggest that this species is in fact a cryptic species complex [5, 12, 13] of which at least four different populations have been identified in Brazil [13]. Some authors have suggested that the taxa of these sibling species could present differences in their vectorial capacities [14–19]. In Sao Paulo state only two populations of this complex has been identified, (S)-9-methylgermacrene-B and Cembrene-1, each one have been associated with areas presenting distinct VL epidemiological patterns [18, 19]. While the Cembrene-1 population is considered native and distributed in the Eastern region of São Paulo state, where no human cases has been recorded, the (S)-9-methylgermacrene-B was identified for the first time in São Paulo in 1997 [18, 19] and is considered a more recently introduced population that occupies Western areas, with occurrence of human cases [18]. In the light of the remarkable epidemiological differences between these regions of São Paulo state, Casanova et al. [18, 19] have suggested that the (S)-9-methylgermacrene-B and Cembrene-1 - chemotype populations could have different vectorial capacities in this state. These differences call attention because in the Northeastern region of Brazil the Cembrene-1 population is abundant and related to human VL cases while that of (S)-9-methylgermacrene-B is rare [20]. These differences could be related to ecological parameters and for this reason studies evaluating aspects of these populations such as the spatial-temporal distribution (abundance, seasonal distribution), natural infection and blood feeding habits, among others, can contribute to the elucidation of the differences between the populations [19] and their role in the transmission of the VL agent in endemic areas.

Climatological variables such as temperature and precipitation can affect biological aspects of hematophagous insects, among these the duration of the immature development and the gonotrophic cycle. Therefore, they influence the spatial-temporal distribution of vectors and produce seasonal patterns [21] and indirectly influences parameters related to the vectorial capacity such as the vector density by host and the biting rate [22]. For this reason, the vector density is a useful indicator in the epidemiological surveillance of vector-borne diseases [23]. Additionally, the vector frequencies are associated with the vegetal cover and density/diversity of the hosts [24, 25], which determine the type and quantity of blood meal sources and the availability of natural shelters, influencing the vector’s contact with a competent host and the prevalence of infection in the vectors. Further, the natural infection estimates in vectors could be used as indicator of transmission intensity to the host, hence determining risk areas.

In Panorama municipality, *Lu. longipalpis* was detected by the first time in 2005 [26] and during the period from 2007 to 2009, 28 human VL cases were notified. According to the to the criteria of the Brazilian Health Ministry to stratify transmission areas for the implementation of prevention and control measures this represent an intense transmission (mean cases number in a period of three years ≥ 4.4 [27]. Based on the number of cases reported [28], between 2009 and 2011 the transmission was moderate (≥ 2.4) and between 2013 and 2015 the transmission was sporadic (≤ 2.4). Considering the endemic character of the VL in this municipality and the epidemiological importance of the vector, this study was designed to investigate the temporal distribution and natural infection of a population of the *Lu. longipalpis* complex in this local to obtain information that may contribute to zoonotic VL surveillance and to the planning of preventive and control measures.

## Methods

### Study area

**Fig. 1:**
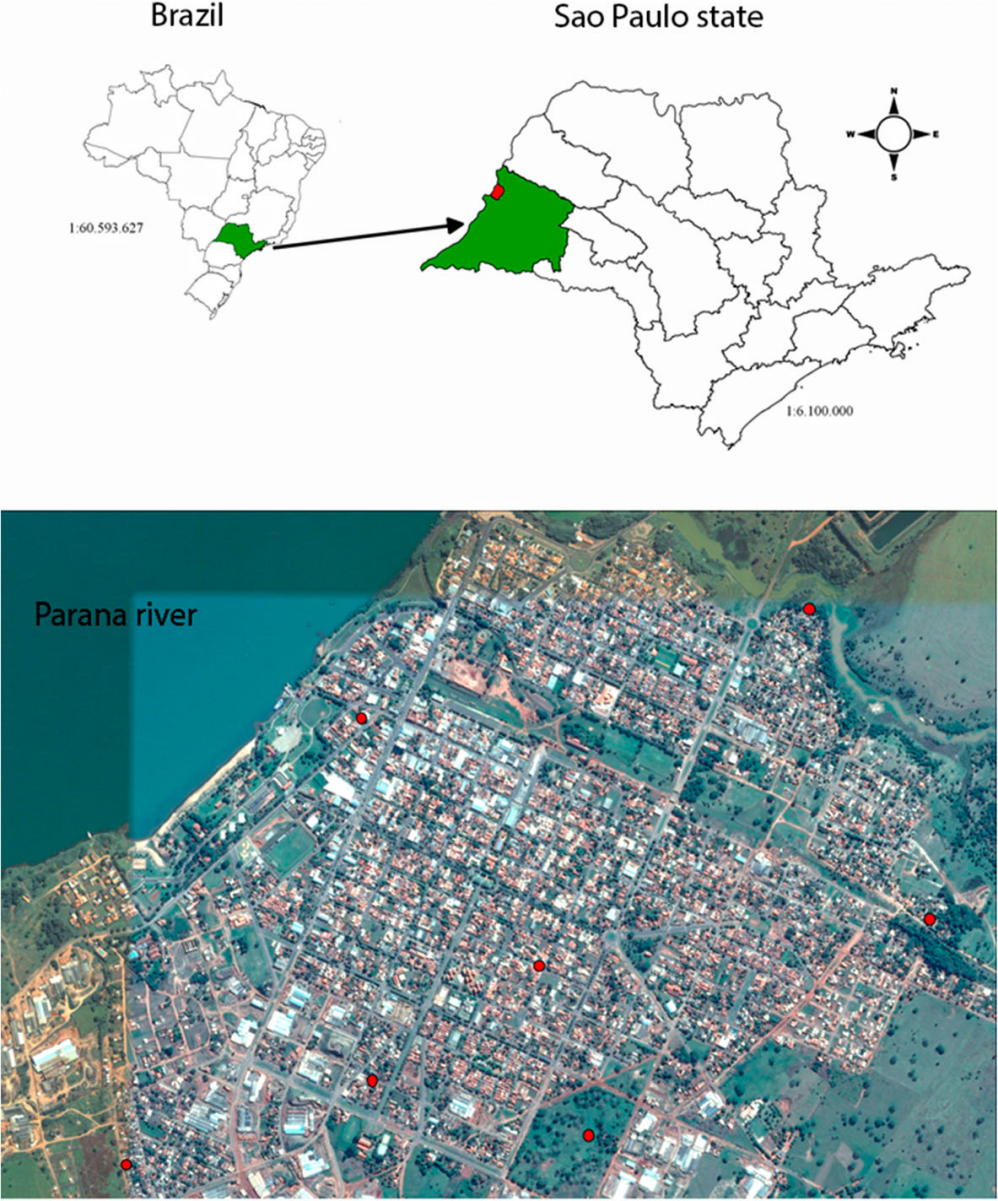
Localization of the sand fly capture points in Panorama Municipality, São Paulo state, Brazil

Panorama municipality is located in the western region of São Paulo state at 21°21′23″S and 51°51′35″W. Its average annual temperature varies between a minimum of 12 °C and a maximum of 35 °C; the rainy season occur from October to March. According to Köppen - Geiger’s classification, the climate is tropical with a dry winter [29]. This region belongs to the Atlantic forest biome, with a population estimated at 14,583 inhabitants, and 95% of them living in urban area [30]. The main economic activities are general services and the ceramics industry. This municipality was selected due to its high annual average numbers of VL human cases and the high canine seroprevalence (41%) [31]. Seven domiciles of the urban area were selected for the entomological survey due to the presence of ecotopes appropriate to sand flies, such as chickencoops or shelters of other domestic animals, and vegetation (Fig. 1).

### Sand fly captures

The captures were performed with automatic light traps installed in the domiciles during two years between July 2012 and June 2014. In each domicile one trap was installed in the porch of the house and another in the peridomicile (animal shelters or vegetation located between 10 and 20 m from the house). The traps were lighted on four consecutive days each month during the first year and on three consecutive days in the second year, in the period between 17:00 and 07:00 h. Among the insects captured a sample of live sand fly females was dissected to investigate natural infection by flagellates in their guts. The other insects were killed by freezing for 10 min and then the samples were screened. The sand flies were counted and registered according to place of capture and collection date. The specimens were stored in ethanol 70%. The head and the terminal part of the abdomen of a sample of the females were severed from the rest of the body, clarified and identified in accordance to the literature [1, 32] and the rest of their bodies were stored for later analysis of natural infection with *Leishmania* sp. by molecular methods. Additionally, the blood feeding state (engorged or non-engorged) and the presence of eggs in the abdomen were observed because this information is an indirect indication of the risk of infection [33].

### Pheromone identification

To identify the *Lu. longipalpis* chemotypes present in the study area specimens captured in 2013 in different places of the Panorama municipality were reared under laboratory conditions to obtain F1 specimens. The natural pheromone test was undertaken on individual (*n* = 6) 5-day-old F1 males. The test was applied according to the method described in Casanova et al. [18].

### Natural infection analysis

A sample of 150 live females were dissected for the parasitological analysis performed as described by Diniz et al. [34]. Another sample of 652 females was grouped into pools for analysis by Nested-PCR. Each pool ranging from between 1 and 10 females consisted of specimens captured in the same place and date.

### DNA extraction

The DNA extraction was performed using a modified method of Brufford et al. [35]. Initially the alcohol used to store the samples was discarded and 20 μl of a lyses solution (Digsol buffer) was added to each pool together with 20 μl of Proteinase K (10 mg/ml) and the samples were incubated for 3 h at 55–60 °C. Thereafter 400 μl of ammonium acetate (4 M) was added, the samples homogenized by vortex for 15 min and centrifuged for 15 min at 13,000 rpm at 10 °C. The supernatant was transferred to another tube and 1 ml of ethanol 100% added and centrifuged at 15,000× *rpm* for 15 min for DNA precipitation. Finally, 900 μl of 70% cold ethanol was added, stirred and the supernatant discarded. After dry, the tube 50 μl of Tris-EDTA buffer was added to re-suspend the pellet.

### Nested PCR for detection and identification of *Leishmania* spp. in the sand flies

To identify the *Leishmania* species in the sand flies, a nested PCR of the SSU rRNA gene (∼561 bp), which corresponds to partial sequence of the variable V7V8 region of the SSU rRNA gene, was carried out according to Noyes et al. [36]. For positive control, we used DNA of females of *Lu. longipalpis* experimentally infected with *Le. infantum.* The PCR-amplified products were sequenced and the generated sequences were submitted to BLAST [37] search in the GenBank database. The sequences obtained were deposited into the GenBank ESTs database with accession numbers MF000384 and MF000385.

### Statistical analyses

Considering the differences in the sampling in the first year (4 nights per month) and the second year (three nights per month), the number of *Lu. longipalpis* specimens collected was standardized as the number of specimens/trap/night. The climatic data were obtained from the meteorological station of Pauliceia municipality located 10 km from Panorama. These data were obtained from the website of the São Paulo state Integrated Center of Agrometeorological Information (CIIAGRO) [38]. A non-parametric analysis of correlation (Spearman’s rank correlation) was undertaken to evaluate the association between the values of specimens of *Lu. longipalpis*/trap/night and the total monthly rainfall 20, 10 days before and on the capture-day and similarly with the average of the temperature values 20 and 10 days before capture and on the capture-day. The analyses were performed by the SPSS V.17 program. In addition, using the Poisson regression, we evaluated the associations between environmental variables and the monthly sand fly density. Thus, the rate ratio (RR) and 95% confidence interval (95% CI) were estimated in both simple and multiple models. The natural infection rate was estimated as the number of positive pools in the total of pools tested, as suggested by Paiva et al. [39].

## Results

### Chemotype identification

In the analysis of the sexual pheromones, all six samples of *Lu. longipalpis* males tested presented the (S) -9-methyl-germacrene B.

### Temporal distribution

A total of 4430 sand flies (1147 females and 3283 males) of six species were captured during the study. *Lutzomyia longipalpis* was the most common species accounting for 97.2% of the specimens captured, with a male/female ratio of 3:1. The number of males and females by species is presented in Table 1. Differences in the male/female ratio were observed in all the months sampled, with higher frequencies of males.

**Table 1:** Number of specimens by species and sex captured in Panorama municipality between July 2012 and June 2014

Species	Females	%	Males	%	Total	%
*Lutzomyia longipalpis*	1087	25.2	3220	74.8	4307	97.22
*Nyssomyia neivai*	35	56.5	27	43.5	62	1.40
*Evandromyia lenti*	19	38.8	30	61.2	49	1.11
*Evandromyia carmelinoi*	2	28.6	5	71.4	7	0.16
*Evandromyia cortelezzi*	3	100.0	0	0	3	0.07
*Brumptomyia brumpti*	1	50.0	1	50.0	2	0.05
Total	1147		3283		4430	100

**Fig. 2:**
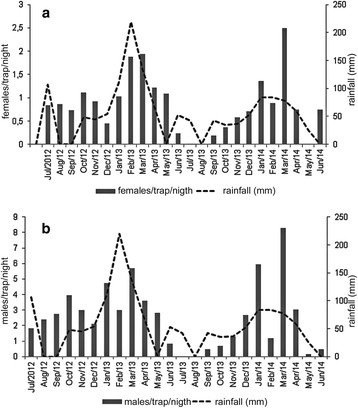
Monthly distribution of females (**a**) and males (**b**) of *Lutzomyia longipalpis* and the total rainfall 20 days before the capture in Panorama (SP) in the period from July 2012 to June 2014

The highest average numbers of sand flies/night/trap were obtained during the rainy season (November-March) with a peak in both male and female abundance in March of both years investigated (Table 2, Fig. 2). With the exception of July and August 2013, *Lu. longipalpis* was collected in all the months sampled with the highest abundance occurring in the period from January to March in both years sampled (Fig. 2).

**Table 2:** Monthly values of males and females of *Lutzomyia longipalpis*, minimum and maximum values of temperature and total values of rainfall in the study area

Month/Year	Temp_max_ 20^a^	Temp_min_ 20^b^	Total rainfall 20^c^	Total no. of specimens		No. of specimens/trap/night	
				Females	Males	Females	Males
Jul/12	27.1	14.5	106.7	53	115	0.83	1.8
Aug/12	30.5	15.9	0.0	55	153	0.86	2.39
Sep/12	32.2	15.7	0.0	35	131	0.73	2.73
Oct/12	31.3	17.7	48.0	71	253	1.11	3.95
Nov/12	33.9	20.9	44.7	59	191	0.92	2.98
Dec/12	34.7	21.8	54.4	28	134	0.44	2.09
Jan/13	32.7	21.3	110.4	49	227	1.02	4.73
Feb/13	32.2	20.8	219.5	120	192	1.88	3.0
Mar/13	32.5	20.8	134.7	124	364	1.94	5.69
Apr/13	29.1	17.8	65.5	78	229	1.22	3.58
May/13	30.0	14.6	1.3	69	180	1.08	2.81
Jun/13	28.7	15.9	52.7	15	53	0.23	0.83
Jul/13	28.6	14.7	42.2	0	0	0	0
Aug/13	30.2	11.9	0	0	0	0	0
Sep/13	33.3	17.1	42.1	8	20	0.19	0.48
Oct/13	32.8	19.2	34.8	15	29	0.36	0.69
Nov/13	33.6	19.9	36.2	24	54	0.57	1.29
Dec/13	35.3	21.1	52.2	30	112	0.71	2.67
Jan/14	33.5	20.4	83.7	57	250	1.36	5.95
Fev/14	35.5	20.7	83.9	37	49	0.88	1.17
Mar/14	33.3	20.2	77.8	104	348	2.49	8.29
Apr/14	30.3	18.7	59.1	31	127	0.74	3.02
May/14	31.6	16.2	25.1	0	4	0	0.14
Jun/14	30.2	14.8	0	10	20	0.74	0.48

^a^Average maximum temperature 20 days before the capture

^b^Average minimum temperature 20 days before the capture

^c^Total value for precipitation 20 days before the capture

A positive and significant correlation was detected between the numbers of males (specimens/trap/night) and the minimum temperature values 10 days before the collection (*r =* 0.47, *P* = 0.02). A positive association with the minimum temperature and the total rainfall 20 days before the collection was observed for both sexes (Table 3).

**Table 3:** Values of the Spearman’s correlation between the climatological variables and the frequency of males and females of *Lutzomyia longipalpis*

Variable	Males/trap/night		Females/trap/night	
	Spearman’s coefficient	Significance level (95%)	Spearman’s coefficient	Significance level (95%)
PT10D^a^	0.21	0.32	0.30	0.14
TMX10D^b^	0.26	0.20	0.03	0.90
TMN10D^c^	0.47^*^	0.02	0.39	0.05
PT20D^d^	0.52^*^	0.01	0.63^*^	< 0.001
TMX20D^e^	0.27	0.19	0.18	0.38
TMN20D^f^	0.50^*^	0.01	0.44^*^	0.03

*P < 0.05; values statistically significant

^a^Total rainfall 10 days before the capture

^b^Average maximum temperature 10 days before the capture

^c^Average minimum temperature 10 days before the capture

^d^Total rainfall 20 days before the capture

^e^Average maximum temperature 10 days before the capture

^f^Average minimum temperature 10 days before the capture

In the analysis of the association of these variables, we observed a biological gradient between environmental variables and total sand fly abundance. Therefore, we have represented these associations by obtaining rate ratios of sand fly counts per unit of both temperature and rainfall. Thus, we estimated, in a multiple model, that sand fly frequencies increase about 22% for each 50 mm of cumulative rainfall (Rate ratio, RR: 1.22; 95% CI: 1.18–1.25); and about 12% for each degree of minimal temperature (RR: 1.12; 95% CI: 1.1–1.14).

Among the 733 females observed to evaluate the blood feeding state and the presence of eggs, 468 were captured in the peridomicile and 265 in house porches. Among those captured in the peridomicile, 75 females (16.0%) were engorged, 7 (1.5%) were engorged and carrying eggs and 35 (7.5%) had only eggs. Between the females captured in the porches, 21 were engorged (7.9%), one (0.4%) was engorged and carrying eggs and 13 (4.5%) only had eggs.

### Natural infection

In the samples of females dissected (*n* = 150) no flagellates were detected in the guts. By using the nested PCR method, a total of 308 pools were analyzed, of which 251 were of *Lu. longipalpis*, 25 of *Ny. neivai*, 18 of *Ev. lenti*, 12 of *Ev. cortelezzii* and two of *Brumptomyia* sp. Only three pools of *Lu. longipalpis* were positive for *Leishmania* sp.; of these, two were females captured in the porch of the same domicile, one with 10 females captured in March 2013 and the second with 6 females captured in June 2014; in both pools there was a female with some remaining eggs in its abdomen. The third positive sample was represented by one female without eggs in its abdomen captured in January 2014 in the peridomicile of a different domicile.

In the alignment of the sequences obtained from the positive samples, a correspondence was observed with those of *Le. infantum* deposited in the GenBank and which clearly differed from those of other species of the subgenera *Leishmania* and *Viannia*. Thus, a minimum infection rate of 1.2% of *Lu. longipalpis* with *L. infantum* was estimated*.*

## Discussion

The first step for planning an effective vector-born disease control program is the identification of the species, which could be acting as vectors and their spatial-temporal distribution in order to identify the periods of highest risk of transmission [23]. Our results of chemotypes analyses of the male samples of Panorama municipality shown to contain the pheromone (S)-9-methylgermacrene-B exclusively. This results is concordant with previous reports in others ten municipalities of the West region of Sao Paulo state [18]. The presence of *Lu. longipalpis* at all the points sampled was observed during the 22/24 months of captures with an average male/female ratio of 3:1. The high abundance of this sand fly observed in the study could be explained by the characteristics of the landscape of the area studied including peridomiciles with some fruit trees and domestic animal shelters, which offer conditions favorable to the development of immature forms of *Lu. longipalpis* and where the adults can find sources of blood and sugar [40, 41]. The predominance of males might be related to the presence of hosts and as a response to aggregation pheromones liberated by males to attract conspecific specimens on the animals as observed in other *Lu. longipalpis* population in VL focus in Brazil [42, 43] and also to the more intense activity of the males in the animal shelters [42] where the light traps were installed, increasing their capture chances.

Our results suggest a predominance of nulliparous females (75.0–85.0%) in the ecotopes evaluated among the females captured and analyzed to evaluate blood feeding and the state of egg development. However, there was a remarkably high proportion of females carrying eggs in the peridomicile (7.5%) and in the porches (4.5%). This information suggests the circulation of females with complete digestion of the blood meal near the domicile and with potential to take a blood meal, indicating a risk to the human and canine populations [33].

We also observed that in the study area, the high density of *Lu. longipalpis* population occurred in the rainy season with the highest abundance in the period from October to March. These observations are consistent with the results obtained in other VL foci in Brazil for other populations of the *Lu. longipalpis* complex [44–46]. This distribution could be corroborated by the observation of a positive and significant association between the abundance of *Lu. longipalpis* and the values of total rainfall 20 days before the capture and between values of minimum temperature. In the analysis of the association between the climatic variables and sand fly abundance, it was estimated that the abudance of *Lu. longipalpis* increased 22% for each 50 mm of increased rainfall and 11% for each degree increase in minimum temperature. Our results reinforce those of previous studies reporting an effect of rainfall on the distribution of this species. This fact could be related to the maintenance of soil humidity favorable to the development of immature forms [47], while the temperature could influence parameters such as the duration of the immature cycle, blood-feeding rate and the duration of the gonotrophic cycle [25, 48, 49]. In Dracena (SP) municipality located at 30 km from Panorama where the same chemotype of *Lu. longipalpis* was identified [18], the same pattern of distribution in a long-term study using automatic light traps has been observed [50]. Thus, our results suggest that the risk of transmission of *L. infantum* in Panorama municipality increases in the rainy season. Therefore, the present results may serve as a guide for the control strategies recommended by the Health authorities focused on the vector, such as the application of residual insecticides [27] and environmental management measures, and also to the evaluation and implementation of large-scale programs such as the use of collars impregnated with insecticides and vaccines to protect dog populations.

In the present study we also analyzed the natural infections with *Leishmania* sp. of specimens of five species (91.9% of *Lu. longipalpis*) by parasitological and molecular tests. In the parasitological analyses by dissection, no flagellates were observed in the gut of the females examined. This result could be related to the higher activity of nulliparous females seeking blood-feeding sources (> 75%), captured in the CDC traps. Perhaps the use of other methods to capture engorged females in the shelters could result in rates closer to those found by other authors, which vary between 0.2 and 7.14% [51, 52].

To evaluate a natural infection of females samples by *Leishmania* species, the molecular analysis by nested PCR was used. More recently molecular methods have been supporting entomological surveillance activities due to their higher sensitivity, the specificity of parasite identification and especially the possibility of analyzing a greater number of specimens [53]. Among the species captured in the focus, only females of *Lu. longipalpis* were positive to infection with *Le. infantum* rendering a minimum infection rate of 1.2%. Using molecular methods, infection rates ranging between 0.25 and 3.9% have been reported in other foci of VL in Brazil [39, 53–55]. These differences could be related to variations in the sensitivity and specificity of the method for identification of the parasite. However, it is emphasized that natural infection in vectors is likely to vary between localities because it depends on factors such as the capture method, host abundance and parasite prevalence in the focus, among others.

The low infection rate in the sand flies observed in this study contrasts with the high canine prevalence (around 40%) in the study area in recent years [26, 31], that increase the risk of infection to humans. However, the identification of females carrying *Le. infantum* DNA captured in two (28.5%) of the seven domiciles sampled, demonstrate a co-circulation of infected host and competent vectors with potential risk to the humans and reinforces the necessity of control vector measures.

The domiciles where potentially infective females were captured were characterized by the presence of abundant blood-feeding sources (hens, dogs, pigs), a factor that favors vector density and the maintenance of the transmission cycle and shows a distribution pattern indicative of a hot-spot [6]. Among the pools with specimens infected with *Le. infantum,* two were formed of females captured in the porch of the domicile and one in the peridomicile, suggesting the contact of competent vectors and some infected host near human domiciles, with the presence of all the components of the nidus of infection [25]. The identification of periods of increased *Lu. longipalpis* abundance and of the circulation of infected females reinforces the necessity of investigation of other vector bionomic parameters related to vectorial capacity, such as survival, vector competence and the density per host among others [22], that contribute to the development of prevention and control measures for VL by health authorities.

## Conclusions

Our results emphasize the vectorial potential of the (S)-9-methyl germacrene B population, whose chemotype has been spreading throughout the western municipalities of São Paulo state, with serious implications for the emergence of VL in this area [18], and for the first time describe ecological parameters for this chemotype of the *Lu. longipalpis* complex. This information constitutes the basis for future evaluation of the vector and dog control measures in this highly endemic VL focus. In view of the fact that Panorama municipality belongs to the most intense VL transmission region in São Paulo, our study contributes ecological data to increase knowledge on sand fly ecology in this region. Further studies evaluating parameters of the vectorial capacity such as survival, host-biting rate, blood feeding habit of this *Lu. longipalpis* chemotype are necessary to the comprehension of the VL dynamics, as well as to evaluate the existence of differences in the vectorial capacity of this chemotype as suggested by some studies [18, 19].

## Abbreviations

DNA: Deoxyribonucleic acid
PCR: Polymerase chain reaction
VL: Visceral leishmaniasis
